# Perioperative Outcomes of Robotic Versus Conventional Total Laparoscopic Hysterectomy in Surgically Complex Cases: A Propensity Score-Matched Study

**DOI:** 10.3390/jcm15072689

**Published:** 2026-04-02

**Authors:** Kyung Jin Eoh, Hyewon Hur, Joo Hyun Park

**Affiliations:** 1 Department of Obstetrics and Gynecology, Yongin Severance Hospital, Yonsei University College of Medicine, Yongin 16995, Republic of Korea; kjeoh2030@yuhs.ac (K.J.E.); hwhur201@yuhs.ac (H.H.); 2 Institute of Women’s Life Medical Science, Yonsei University College of Medicine, Seoul 03722, Republic of Korea

**Keywords:** hysterectomy, robotic surgical procedures, laparoscopy, blood loss, surgical, propensity score

## Abstract

**Objective**: To compare perioperative outcomes between robotic and conventional total laparoscopic hysterectomy in terms of operative time, intraoperative blood loss, and postoperative recovery in surgically complex cases. **Methods**: This retrospective cohort study included patients that underwent total laparoscopic hysterectomy between 2020 and 2022. As robotic surgery was preferentially applied to more complex cases in an effort to minimize the risk of open conversion, propensity score matching based on uterine weight and history of abdominal surgery was performed. The normality of continuous variables was assessed using the Shapiro–Wilk test; non-normally distributed variables are reported as median [interquartile range] and compared using the Mann–Whitney U test. Multivariate linear regression with log-transformed estimated blood loss was conducted to evaluate the independent association of surgical approach with hemostatic outcomes. **Results**: After 1:1 matching, 93 patients were analyzed per group. Robotic surgery was associated with longer operative time but lower estimated blood loss when compared with conventional laparoscopy. Postoperative hemoglobin decline, length of hospital stay, and complication rates were comparable between groups. In multivariate analysis, uterine weight and operative time were the primary determinants of estimated blood loss; surgical approach showed a modest, independent association with lower log-transformed estimated blood loss after adjustment for these factors. **Conclusions**: Robotic and conventional total laparoscopic hysterectomy demonstrated comparable perioperative safety profiles with different operative trade-offs. Observed differences in estimated blood loss reflect complex interactions between surgical difficulty, operative time, and instrumentation rather than inherent platform superiority. These findings support an individualized approach to surgical modality selection based on case complexity, to minimize risk of intraoperative complication leading to open conversion.

## 1. Introduction

Total laparoscopic hysterectomy (TLH) is widely accepted as the standard minimally invasive approach for hysterectomy, offering faster recovery and reduced postoperative morbidity when compared with open surgery [1]. However, conventional laparoscopy can be technically challenging in patients with large uteri, pelvic adhesions, or prior abdominal surgery, where limited instrument articulation and ergonomics may increase operative difficulty and intraoperative blood loss [2].

Robotic laparoscopy was introduced to address these limitations by providing enhanced three-dimensional visualization, articulated instruments, and improved precision in dissection and hemostasis. Moreover, surgeon fatigue may be minimized providing better ergonomics, with less clashing among tools and the target organ for difficult surgical cases [3,4]. These technical advantages may be particularly beneficial in complex hysterectomy cases, leading to the increased adoption of robotic TLH in patients with anticipated surgical difficulty [5].

Previous comparative studies and meta-analyses have reported inconsistent results regarding perioperative outcomes between robotic and conventional total laparoscopic hysterectomy [6,7]. Several randomized controlled trials and large observational studies have found no significant difference in estimated blood loss or complication rates, while others have reported modest advantages with robotic assistance in selected patient populations [7,8]. A 2023 systematic review and meta-analysis by Lenfant et al. identified that differences across studies were substantially attributable to case-mix heterogeneity and selection bias, with robotic surgery frequently applied to more complex cases involving larger uteri or anticipated adhesions [3]. When robotic surgery is preferentially selected for anticipated complexity, two important sources of bias that emerge are as follows: first, standard group comparisons conflate the effect of surgical complexity with the effect of the surgical platform; second, surgeons who choose robotic surgery for difficult cases may exercise greater intraoperative caution—independent of the platform—potentially influencing hemostatic outcomes through behavioral rather than technical mechanisms. Propensity score matching (PSM) can address the former but cannot fully resolve the latter [8].

Studies specifically focused on cases with large uteri have suggested that robotic assistance may offer hemostatic advantages in this context, though evidence remains limited by small sample sizes and heterogeneous definitions of operative complexity [9,10]. Among the few propensity-matched analyses available, most have matched on broad demographic variables rather than direct markers of surgical complexity such as uterine weight and prior abdominal surgery history, potentially leaving substantial residual confounding [8].

In this study, we compared perioperative outcomes between Robotic and Conventional TLH using a propensity score-matched design that specifically accounts for surgical complexity by matching uterine weight and history of prior abdominal surgery. We additionally performed multivariate regression analyses and addressed the potential role of confounding by surgical indication in the interpretation of our findings. Rather than testing a directional hypothesis of robotic superiority, we aimed to characterize the operative trade-offs associated with each approach in the context of complex hysterectomy.

## 2. Materials and Methods

### 2.1. Study Population and Design

This retrospective cohort study was conducted at Yongin Severance Hospital, Yonsei University College of Medicine, Yongin, Republic of Korea. We reviewed the medical records of patients who underwent total laparoscopic hysterectomy for benign uterine diseases (such as uterine leiomyoma and adenomyosis) between March 2020 and December 2022. The study protocol was approved by the Institutional Review Board (IRB) of Yongin Severance Hospital (Approval No. 2024-0535-002). Due to the retrospective nature of this study, the requirement for informed consent was waived.

Patients were divided into two groups according to the surgical approach: the robotic total laparoscopic hysterectomy (R-TLH) group and the conventional total laparoscopic hysterectomy (L-TLH) group. The exclusion criteria were as follows: (1) malignancy confirmed by pathology, (2) conversion to laparotomy due to unexpected malignancy or massive bleeding not related to the surgical difficulty itself, and (3) concomitant surgeries other than adnexal surgery (e.g., cholecystectomy, sacro-colpopexies for pelvic organ prolapse).

Surgical approach selection followed institutional practice patterns. Robotic surgery was preferentially selected when uterine weight was expected to exceed approximately 500 g or the level of uterine fundus approaches the umbilicus on pelvic examination, when extensive pelvic adhesions were anticipated (e.g., prior abdominal surgery, endometriosis, significant bowel disease), or when the surgeon’s clinical assessment indicated high operative complexity. Conventional laparoscopy was used for cases of lower anticipated complexity. This systematic, non-random allocation is the primary motivation for propensity score matching and represents the central source of potential confounding in this study.

### 2.2. Surgical Procedures

All surgeries were performed by a single experienced gynecologic surgeon, well beyond the learning curve, having performed around 1400 robotic and 5000 laparoscopic gynecologic procedures. During the study period, robotic procedures were performed using either the da Vinci Xi or da Vinci SP platform (Intuitive Surgical, Sunnyvale, CA, USA) depending on system availability and case characteristics. Although both platforms provide articulated instruments and three-dimensional visualization, potential platform-related differences in surgical performance cannot be completely excluded. With the patient in Trendelenburg and lithotomy position, a central camera port was placed at the umbilicus, with accessory ports positioned according to the surgeon’s preference. The robotic arms for the da Vinci Xi multiport cases were placed 8 cm lateral and 2 cm caudal to the umbilical port. A central camera port was placed at the umbilicus, with accessory ports positioned according to the surgeon’s preference. The robotic platform provided stable three-dimensional visualization and articulated instruments, facilitating precise dissection and atraumatic tissue handling with least clashing between the tools and the uterus even in cases with large uterine sizes.

In the L-TLH group, standard multiport laparoscopic techniques were used with rigid instruments. Due to the limited range of motion of conventional laparoscopic tools, counter-traction and manipulation were technically more demanding in cases with enlarged uteri.

In both groups, uterine manipulation was performed using the Rumi II system. The uterus was removed vaginally after encasing the specimen within a laparoscope bag followed by manual morcellation with a surgical blade, and the vaginal cuff was closed intracorporeally using absorbable sutures.

### 2.3. Data Collection and Outcome Measures

Patient baseline characteristics included age, body mass index (BMI), parity, history of abdominal surgery (including cesarean section), and American Society of Anesthesiologists (ASA) physical status score. Uterine weight was measured immediately after removal in the operating room or calculated based on the pathology report.

Perioperative outcomes included total procedure time, console time (for R-TLH), estimated blood loss (EBL), change in hemoglobin (Hb) level (preoperative vs. postoperative day 1), length of hospital stay, and intraoperative or postoperative complications. Estimated blood loss was measured by collecting intraoperative bleeding in a graduated bottle with suction–irrigation, and the volume of normal saline used was substracted after every procedure.

### 2.4. Statistical Analysis

All analyses were performed using R version 4.4.2 (R Foundation for Statistical Computing, Vienna, Austria). Normality of continuous variables was assessed using the Shapiro–Wilk test. All key continuous variables (age, BMI, uterine weight, parity, EBL, operative time, hospital stay, and Hb decline) were non-normally distributed (Shapiro–Wilk *p* < 0.05 for all). These variables are therefore presented as median [interquartile range (IQR)] and compared using the Mann–Whitney U test. Categorical variables are expressed as numbers (percentages) and compared using the Chi-square test or Fisher’s exact test.

To verify that the operating surgeon had achieved stable proficiency for both surgical platforms prior to the comparative analysis, cumulative sum (CUSUM) analysis of operative time was performed separately for R-TLH and L-TLH [11]. For each approach, operative times were ordered chronologically by case sequence, and the CUSUM was calculated as the cumulative deviation of each case’s operative time from the overall procedural mean. The learning curve transition point was defined as the case number at which the CUSUM curve reached its maximum value, representing the inflection from above-average to below-average operative times. Pre- and post-transition operative times were compared using the Mann–Whitney U test. Plateau stability was assessed by comparing operative times in the final tercile of cases against the immediately preceding comparable interval using the same test. A 7-case centered moving average was additionally computed and displayed to facilitate the visual assessment of temporal trends. Cases with missing or zero operative time values were excluded from this analysis.

Propensity scores were estimated using logistic regression including uterine weight and history of abdominal surgery as covariates, reflecting the primary clinical drivers of surgical approach selection. Patients were matched 1:1 using nearest-neighbor matching with replacement and a caliper of 0.2 standard deviations of the logit of the propensity score. Matching with replacement was used to improve match quality given the smaller and more homogeneous robotic cohort. Covariate balance was assessed using the standardized mean difference (SMD), with an SMD of <0.1 considered adequate. Residual imbalances in age and parity after matching were incorporated into multivariate analyses. A *p*-value of <0.05 was considered statistically significant.

Multivariate linear regression with log-transformed EBL was performed to evaluate independent predictors of blood loss. Covariates included surgical approach, uterine weight, total operative time, age, parity, and history of prior abdominal surgery. Adjusted beta coefficients with 95% confidence intervals are reported.

## 3. Results

### 3.1. Patient Characteristics Before and After Propensity Score Matching

**Figure 1:**
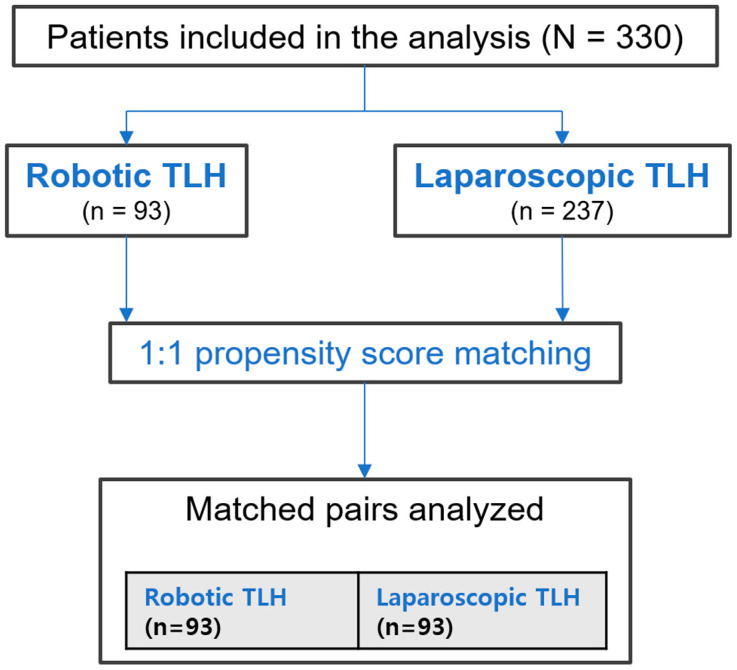
The flow diagram of patient selection and propensity score matching. A total of 336 patients were assessed for eligibility, of whom 6 were excluded due to missing data. Overall, 330 patients were included in the analysis, comprising 93 patients who underwent robotic total laparoscopic hysterectomy and 237 patients who underwent conventional laparoscopic hysterectomy. Propensity score matching was performed using uterine weight and history of abdominal surgery, resulting in a 1:1 matched cohort of 93 pairs for the final analysis.

A total of 330 patients were included; of these patients, 93 underwent R-TLH and 237 underwent L-TLH (Figure 1). Before PSM, uterine weight was significantly greater in the robotic group, consistent with the institutional practice of selecting robotic surgery for more complex cases. Other baseline characteristics including age, BMI, parity, and history of prior surgery were comparable between groups before matching. No patients in either group required perioperative blood transfusion.

After 1:1 PSM, 93 matched pairs were analyzed. Uterine weight was well balanced after matching (SMD < 0.1). BMI and history of prior surgery remained comparable. However, patients in the conventional group were older (50.0 [46.0–52.0] years vs. 48.0 [45.0–51.0] years, *p* = 0.012) and had higher parity after matching (*p* < 0.001). These residual imbalances, which reflect the constraints of matching with replacement and the available case distribution, were addressed in multivariate analyses (Table 1).

**Figure 2:**
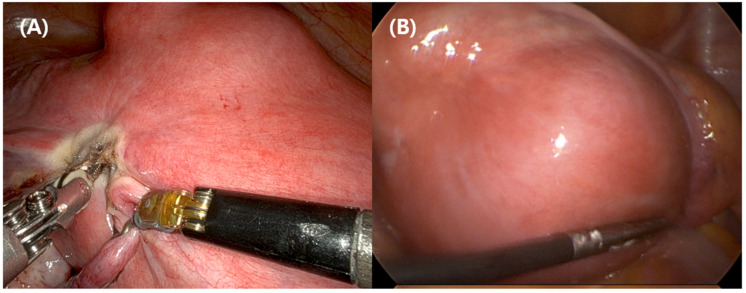
The representative intraoperative views of robotic and conventional total laparoscopic hysterectomy in cases with large uteri. (**A**) Robotic total laparoscopic hysterectomy (R-TLH) performed using the da Vinci Xi system in a 44-year-old patient with a uterine specimen weight of 729.1 g and dimensions of 22.3 × 21.7 × 18.2 cm, demonstrating enhanced instrument articulation and stable three-dimensional visualization that facilitate atraumatic tissue handling despite limited pelvic working space. (**B**) Conventional total laparoscopic hysterectomy (L-TLH) performed in a 50-year-old patient with a uterine specimen weight of 498 g and dimensions of 17.1 × 17.0 × 14.2 cm, using rigid instruments, where counter-traction and fine manipulation are more challenging due to instrument clashing and restricted range of motion in the setting of large uterine size. All images are representative cases from the study cohort.

Representative intraoperative images illustrating technical differences between the two approaches in cases with large uteri are shown in Figure 2.

### 3.2. Surgeon Learning Curve Analysis

The CUSUM analysis of operative time was performed to confirm that the surgeon had achieved stable proficiency for both platforms throughout the study period (Supplement Figure S1). For R-TLH (n = 93), the CUSUM transition point was identified at case 41, with median operative time decreasing from 167 min [IQR = 150–197] before the transition to 150 min [IQR = 128–166] after (10.5% reduction; *p* = 0.001); operative time showed no further significant decline beyond this point (last 30 vs. preceding 30 cases: *p* = 0.953), confirming plateau-level performance. For L-TLH (n = 237), the transition occurred at case 110, with a comparable reduction from 106 min [IQR = 89–131] to 87 min [IQR = 74–102] (17.9%; *p* < 0.001), followed by stable performance thereafter (*p* = 0.544). Both transition points fell within the first half of each procedural series (46% and 47% of total cases, respectively) and converged temporally in late 2021, indicating that proficiency for both approaches had been established well before the majority of cases contributing to the comparative analysis. The modest absolute magnitude of improvement—18 and 19 min, respectively—is consistent with technical refinement and enhanced surgical teamwork rather than primary skill acquisition, confirming that the surgeon was well beyond the learning curve for both platforms during the study period.

### 3.3. Operative Outcomes After Propensity Score Matching

Total operative time was significantly longer in the robotic group (155 [135–183] min vs. 101 [91–137] min, *p* < 0.001) (Table 2). This difference reflects inherent workflow distinctions between the two platforms, including system setup, docking, and instrument exchange steps that are unique to robotic procedures and are not components of the surgeon’s active dissection time.

EBL was lower in the robotic group than in the conventional group (*p* = 0.016; Table 2). However, this difference was not reflected in postoperative hemoglobin decline (2.1 [1.4–2.8] g/dL vs. 1.9 [1.4–2.5] g/dL, *p* = 0.582), a more objective and less subjectively influenced measure of intraoperative blood loss. Length of hospital stay was comparable between groups (5.0 [5.0–5.0] vs. 5.0 [5.0–5.0] days, *p* = 0.377). Complications were rare and did not differ significantly between groups. No cases of open conversion occurred in either group.

In multivariate linear regression, greater uterine weight (adjusted β = 0.11 per 100 g, 95% CI = 0.06–0.16, *p* < 0.001) and longer operative time (adjusted β = 0.09 per 30 min, 95% CI = 0.03–0.15, *p* = 0.004) were the strongest independent predictors of higher EBL, underscoring that hemostatic outcomes are primarily driven by surgical complexity and operative duration rather than platform choice (Table 3). After adjustment for these factors, robotic surgical approach retained a modest, independent association with lower log-transformed EBL (adjusted β = −0.28, 95% CI = −0.44 to −0.12, *p* = 0.001). Age, parity, and history of prior surgery were not independently associated with EBL.

## 4. Discussion

Validating whether the robotic platform is beneficial for minimizing the risks for complex hysterectomy cases with huge uterine volume or anticipated adhesions is an important goal to pursue in an era where the advancement of minimally invasive techniques is quite pronounced. In this propensity score-matched retrospective analysis, robotic TLH was associated with lower intraoperative estimated blood loss and longer operative time when compared with conventional TLH, while postoperative hemoglobin decline, length of hospital stay, and complication rates were comparable between groups. Importantly, multivariate analysis demonstrated that uterine weight and operative time were the dominant determinants of EBL, with surgical approach showing only a modest association after adjustment. Open conversion and remarkable perioperative complications were negligible for both modalities, even with significantly larger uterine sizes in the robotic-TLH group. These findings suggest that observed perioperative differences reflect a complex interplay of surgical complexity, operative strategy, and instrumentation rather than unidirectional platform superiority.

The technical rationale for potential hemostatic advantages of robotic surgery is well established. Robotic platforms provide stable three-dimensional visualization, tremor filtration, and articulated instruments that may facilitate precise tissue dissection and secure vascular control, particularly during technically demanding steps such as ligation of uterine vessels and management of enlarged, adherent uteri [12,13]. These features may be particularly relevant in cases with large uteri where conventional rigid instruments face mechanical disadvantages due to limited working space and restricted tool motion, and forced traction to the uterus precipitate to increased tissue injury and bleeding. Since the uterus is anatomically in close proximity to the bladder, the ureters and the bowel, unexpected injuries to adjacent structures increase with uterine size [9,10,13].

However, interpretive limitations of this study—and of observational surgical research more broadly—warrant explicit discussion: confounding by surgical indication due to a retrospective study design. In complex hysterectomy cases, finding a modality which would significantly decrease intraoperative bleeding has significant value. However, as robotic surgery was preferentially allocated to anticipated complex cases, surgeons selecting this approach for difficult patients may also have exercised greater intraoperative caution and deliberate hemostatic technique, independently of the platform’s technical capabilities. This behavioral confounding—wherein the surgeon’s decision framework, rather than the instrument itself, drives outcomes—cannot be eliminated by propensity score matching, which can only balance measured preoperative characteristics [14,15]. It is therefore not possible to attribute the observed difference in EBL exclusively to the technical properties of robotic surgery. Our findings are more appropriately interpreted as demonstrating an association between surgical approach and EBL in the complex hysterectomy context, rather than a causal hemostatic advantage.

Notably, the observed reduction in EBL in the robotic group was not reflected in postoperative hemoglobin decline, which was comparable between groups (2.1 [1.4–2.8] vs. 1.9 [1.4–2.5], *p* = 0.582). This discordance warrants consideration. Estimated blood loss is a subjective, operator-dependent measure subject to systematic bias, whereas hemoglobin decline provides a more objective surrogate for true blood loss [16,17]. The absence of a corresponding difference in hemoglobin decline tempers the clinical significance of the EBL finding and should be prominently considered when interpreting the comparative results [18]. This discrepancy may also reflect measurement variability in EBL estimation, particularly in complex cases where irrigation volumes are large.

The longer operative time observed with robotic surgery is consistent with that reported in the prior literature and reflects not only the surgeon’s active dissection time but also setup, docking, and instrument exchange steps inherent to the robotic workflow [12,14,19]. After accounting for case complexity, this difference is a meaningful practical consideration, as it affects operating room throughput and resource allocation [20]. Previous comparative studies including a recent RCT-only meta-analysis by Mirza et al. similarly reported longer operative times with robotic TLH without demonstrating compensatory advantages in other perioperative outcomes, though case-mix heterogeneity across studies limits direct comparison [7].

Our findings support the view that surgical approach selection should be individualized based on case complexity, institutional expertise, and available resources, rather than driven by assumptions of platform superiority [21]. For surgically complex cases—particularly those involving large uteri or anticipated adhesions—either robotic or conventional laparoscopy may be appropriate depending on the surgeon’s training and institutional context [3,8,22]. Patients with relatively large uterine sizes and strong desires to avoid open hysterectomies migrate towards robot myomectomies, whether the conventional L-TLH relatively increases the risk of open conversion in those with huge uterine could not be differentiated in this cohort, with 0% conversion in both groups. Future prospective randomized studies are needed to disentangle platform-specific effects from surgeon behavior and case complexity in determining perioperative outcomes.

This study leaves room for larger multi-center studies in the future. The analysis was performed for an experienced surgeon in both laparoscopic and robotic surgery, removing interobserver variability across different surgical modalities, as proven by the learning curve analysis. However, the outcomes have to be reproducible among different surgeons and centers. The retrospective design introduces potential residual confounding that PSM cannot fully address, particularly with respect to unmeasured determinants of surgical complexity and surgeon behavior as discussed above. Age and parity remained imbalanced after matching, likely reflecting the distribution of available cases and the constraints of matching with replacement; these residual imbalances were incorporated in multivariate adjustments, and the robotic approach retained its modest association with lower EBL. The PSM model was intentionally limited to uterine weight and prior surgery history as the primary drivers of approach selection, potentially omitting other complexity factors. The discordance between EBL and hemoglobin decline findings limits the strength of conclusions regarding hemostatic outcomes. The single-center design with a single highly experienced surgeon, while eliminating inter-surgeon confounding, reduces generalizability to settings with less robotic experience or different case-mix distributions. Additionally, the absence of standardized cost data precluded formal cost-effectiveness analysis, and long-term outcomes were not assessed.

## 5. Conclusions

In this propensity score-matched analysis, robotic and conventional TLH demonstrated comparable perioperative safety profiles in surgically complex cases. The two approaches differed in their operative trade-offs, with robotic surgery associated with longer operative time and lower estimated blood loss. However, the absence of a corresponding difference in postoperative hemoglobin decline, combined with the inherent limitations of retrospective design and the possibility of confounding by surgical indication, precludes the causal attribution of hemostatic differences to the robotic platform itself. Surgical complexity—reflected by uterine weight and operative time—was the primary determinant of intraoperative blood loss in multivariate analysis. These findings support individualized surgical decision making based on case complexity and clinical priorities and underscore the need for prospective randomized studies to clarify the true impact of robotic assistance in complex hysterectomy.

## Figures and Tables

**Table 1:** Patient characteristics.

Characteristic	Before Matching			After Matching		
	R-TLH (*n* = 93)	L-TLH (*n* = 237)	*p*-Value	R-TLH (*n* = 93)	L-TLH (*n* = 93)	*p*-Value
**Age (years)** ^a^	48.0 [45.0–51.0]	47.0 [45.0–50.0]	0.328	48.0 [45.0–51.0]	50.0 [46.0–52.0]	0.012
**BMI (kg/m^2^)** ^a^	23.8 [21.9–26.7]	24.0 [21.6–26.5]	0.945	23.8 [21.9–26.7]	23.5 [22.2–26.9]	0.918
**Ut. Weight (g)** ^a^	688 [522–951]	316 [208–442]	<0.001	688 [522–951]	687 [492–1001]	0.995
**Parity** ^a^	2.0 [1.0–2.0]	2.0 [1.0–2.0]	0.140	2.0 [1.0–2.0]	2.0 [2.0–3.0]	<0.001
**Hx of Surgery**	51 (54.8%)	134 (56.5%)	0.875	51 (54.8%)	49 (52.7%)	0.883

^a^ Non-normally distributed variable (Shapiro–Wilk *p* < 0.05); presented as median [IQR]; compared with the Mann–Whitney U test. R-TLH, robotic total laparoscopic hysterectomy; L-TLH, conventional total laparoscopic hysterectomy; BMI, body mass index; IQR, interquartile range.

**Table 2:** Operative outcomes after PSM.

Outcomes	R-TLH (*n* = 93)	L-TLH (*n* = 93)	*p*-Value
Total Operative Time (min), ^a^ median [IQR]	155 [135–183]	101 [91–137]	<0.001
EBL (mL), ^a^ median [IQR]	200 [80–320]	250 [120–473]	0.016
Postoperative Hb Decline (g/dL), ^a^ median [IQR]	2.1 [1.4–2.8]	1.9 [1.4–2.5]	0.582
Length of Hospital Stay (days), ^a^ median [IQR]	5.0 [5.0–5.0]	5.0 [5.0–5.0]	0.377
Complications, n (%)	1 (1.1%)	0 (0.0%)	1.000
Open Conversion, n (%)	0 (0.0%)	0 (0.0%)	1.000

^a^ Non-normally distributed variable (Shapiro–Wilk *p* < 0.05); presented as median [IQR]; compared with the Mann–Whitney U test. EBL, estimated blood loss; Hb, hemoglobin; IQR, interquartile range; R-TLH, robotic total laparoscopic hysterectomy; L-TLH, conventional total laparoscopic hysterectomy.

**Table 3:** Multivariate linear regression analysis for factors associated with estimated blood loss.

Variable	Adjusted β	95% CI	*p*-Value
R-TLH vs. L-TLH	−0.28	−0.44 to −0.12	0.001
Ut. weight (per 100 g)	0.11	+0.06 to +0.16	<0.001
Total Op time (per 30 min)	0.09	+0.03 to +0.15	0.004
Age (years)	0.01	−0.01 to +0.03	0.28
Parity	−0.04	−0.10 to +0.02	0.18
Hx of surgery	0.07	−0.05 to +0.19	0.25

R-TLH, robotic total laparoscopic hysterectomy; L-TLH, conventional total laparoscopic hysterectomy; CI, confidential interval; Ut., uterus; Op, operation; Hx, history.

## Data Availability

The data supporting the findings of this study are included in the article. Additional information is available from the corresponding author upon reasonable request.

## Supplementary Materials

The following supporting information can be downloaded at: https://www.mdpi.com/article/10.3390/jcm15072689/s1. Figure S1: Surgeon learning curve analysis for robotic (R-TLH, panels A and C) and conv entional (L-TLH, panels B and D) total laparoscopic hysterectomy. Panels A and B display individual op erative times (scatter plots) with 7-case centered moving averages (solid lines) and pre/post-transition me dians (horizontal lines). Panels C and D display CUSUM curves of operative time deviations from the pr ocedural mean; the peak of each curve identifies the learning curve transition point (R-TLH: case 41; L-T LH: case 110). Operative time was significantly shorter after the transition for both approaches (R-TLH: 1 67 vs. 150 min, *p* = 0.001; L-TLH: 106 vs. 87 min, *p* < 0.001), with no further significant reduction beyon d the plateau (R-TLH: *p* = 0.953; L-TLH: *p* = 0.544), confirming stable proficiency throughout the study period. CUSUM, cumulative sum; IQR, interquartile range; R-TLH, Robotic Total Laparoscopic Hysterecto my; L-TLH, Conventional Total Laparoscopic Hysterectomy.

## Abbreviations

The following abbreviations are used in this manuscript:

ASA	American Society of Anesthesiologists
BMI	body mass index
CUSUM	cumulative sum
EBL	estimated blood loss
Hb	hemoglobin
IRB	Institutional Review Board
L-TLH	conventional total laparoscopic hysterectomy
PSM	propensity score matching
R-TLH	robotic total laparoscopic hysterectomy
SD	standard deviation
TLH	total laparoscopic hysterectomy
