# Supplementary material for: Perioperative Outcomes of Robotic Versus Conventional Total Laparoscopic Hysterectomy in Surgically Complex Cases: A Propensity Score-Matched Study
Source: J Clin Med. 2026 Apr 2;15(7):2689. doi: 10.3390/jcm15072689 (PMC13073160; doi:10.3390/jcm15072689)

Supplementary Figure S1. Surgeon learning curve analysis for robotic (R-TLH, panels A and C) and conventional (L-TLH, panels B and D) total laparoscopic hysterectomy. Panels A and B display individual operative times (scatter plots) with 7-case centered moving averages (solid lines) and pre/post-transition medians (horizontal lines). Panels C and D display CUSUM curves of operative time deviations from the procedural mean; the peak of each curve identifies the learning curve transition point (R-TLH: case 41; L-TLH: case 110). Operative time was significantly shorter after the transition for both approaches (R-TLH: 167 vs. 150 min, p = 0.001; L-TLH: 106 vs. 87 min, p < 0.001), with no further significant reduction beyond the plateau (R-TLH: p = 0.953; L-TLH: p = 0.544), confirming stable proficiency throughout the study period. CUSUM, cumulative sum; IQR, interquartile range; R-TLH, Robotic Total Laparoscopic Hysterectomy; L-TLH, Conventional Total Laparoscopic Hysterectomy.

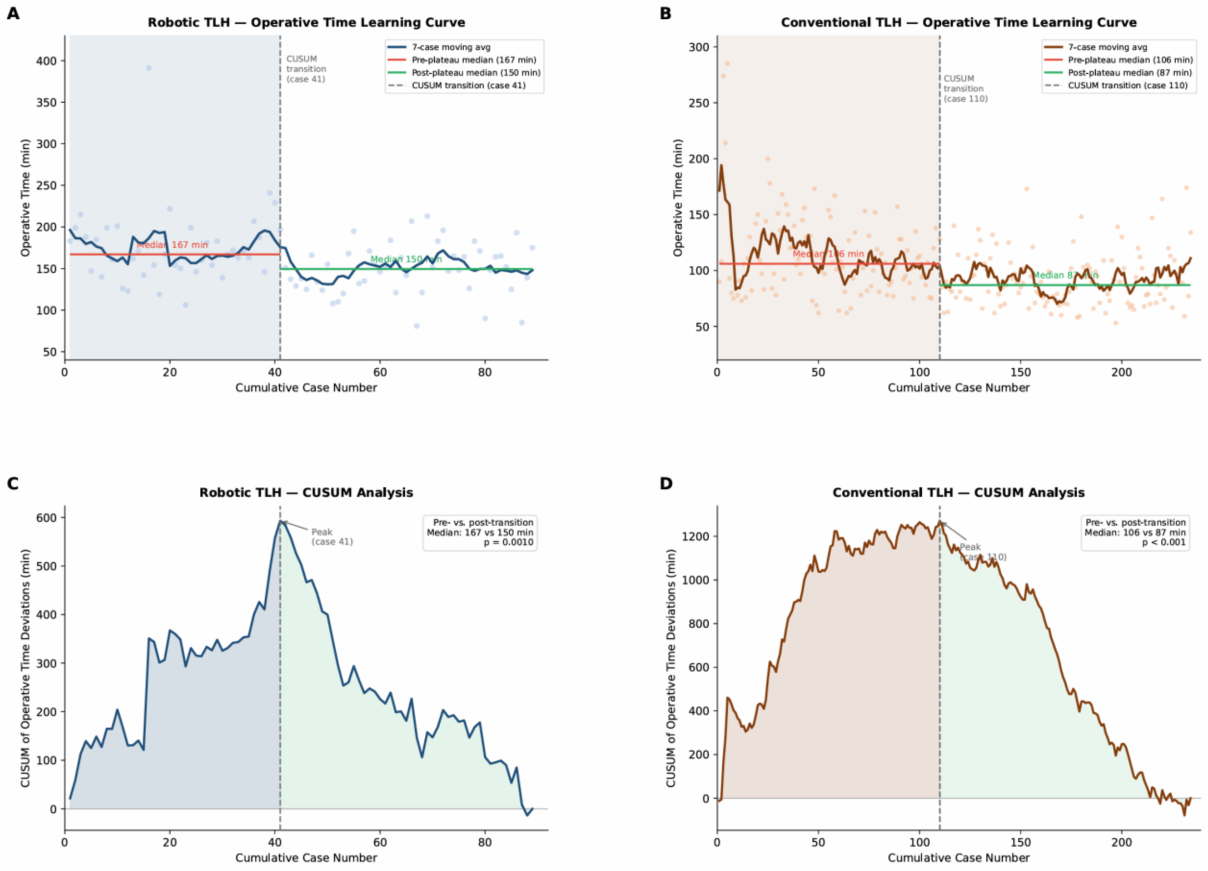

Supplement: Supplementary file 1 [file jcm-15-02689-s001.zip › jcm-4158055-supplementary.pdf]
